# A mass vaccination campaign targeting adults and children to prevent typhoid fever in Hechi; Expanding the use of Vi polysaccharide vaccine in Southeast China: A cluster-randomized trial

**DOI:** 10.1186/1471-2458-5-49

**Published:** 2005-05-18

**Authors:** Jin Yang, Camilo J Acosta, Guo-ai Si, Jun Zeng, Cui-yun Li, Da-bin Liang, R Leon Ochiai, Anne-Laure Page, M Carolina Danovaro-Holliday, Jie Zhang, Bao-de Zhou, He-zhuang Liao, Ming-liu Wang, Dong-mei Tan, Zhen-zhu Tang, Jian Gong, Jin-Kyung Park, Mohammad Ali, Bernard Ivanoff, Gui-chen Liang, Hong-hui Yang, Tikki Pang, Zhi-yi Xu, Allan Donner, Claudia M Galindo, Bai-qing Dong, John D Clemens

**Affiliations:** 1 Division of Bacterial Diseases, Guangxi Center for Disease Control, Nanning, Guangxi, China 80 Taoyuan Road, Nanning 530021, Guangxi, China; 2 International Vaccine Institute, Kwanak P.O. Box 14, Seoul, Korea 151-600; 3 Hechi Anti-epidemic Center, Hechi, Guangxi, China 2 Xinjian Road, Jinchengjiang 54700, Guangxi, China; 4 Vaccines and Other Biologicals, World Health Organization, Geneva, Switzerland; 5 Research Policy and Cooperation, World Health Organization, Geneva, Switzerland; 6 University of Western Ontario, Canada

## Abstract

**Background:**

One of the goals of this study was to learn the coverage, safety and logistics of a mass vaccination campaign against typhoid fever in children and adults using locally produced typhoid Vi polysaccharide (PS) and group A meningococcal PS vaccines in southern China.

**Methods:**

The vaccination campaign targeted 118,588 persons in Hechi, Guangxi Province, aged between 5 to 60 years, in 2003. The study area was divided into 107 geographic clusters, which were randomly allocated to receive one of the single-dose parenteral vaccines. All aspects regarding vaccination logistics, feasibility and safety were documented and systematically recorded. Results of the logistics, feasibility and safety are reported.

**Results:**

The campaign lasted 5 weeks and the overall vaccination coverage was 78%. On average, the 30 vaccine teams gave immunizations on 23 days. Vaccine rates were higher in those aged ≤ 15 years (90%) than in adolescents and young adults (70%). Planned mop-up activities increased the coverage by 17%. The overall vaccine wastage was 11%. The cold chain was maintained and documented. 66 individuals reported of adverse events out of all vaccinees, where fever (21%), malaise (19%) and local redness (19%) were the major symptoms; no life-threatening event occurred. Three needle-sharp events were reported.

**Conclusion:**

The mass immunization proved feasible and safe, and vaccine coverage was high. Emphasis should be placed on: injection safety measures, community involvement and incorporation of mop-up strategies into any vaccination campaign. School-based and all-age Vi mass immunizations programs are potentially important public health strategies for prevention of typhoid fever in high-risk populations in southern China.

## Background

The People's Republic of China has led the use of Vi vaccine as a public health tool to contain typhoid fever in some provinces in which the disease is endemic. With Vi vaccine proven to be efficacious in clinical trials [1,2], in 1989 the Lanzhou Institute of Biological Products began planning for the production of Vi vaccine in cooperation with the original developers [3]. The production followed the specifications published by the World Health Organization (WHO) [4]. In 1995, 2 large-scale licensing randomized, placebo-controlled trials in China of this locally produced vaccine demonstrated its safety and protective efficacy (71% and 69% protection 12 and 19 months after vaccination, respectively) [5], [6]. During an outbreak in 1999, the vaccine was found to be 71% effective [7], similar to the efficacy results reported in phase III trials.

Vi vaccine is currently produced by 6 vaccine institutes in China. Some provincial and district governments have encouraged its use through school-based campaigns financed with reasonable user fees (e.g., US $0.30 to $0.60 per dose). In 1996, the Guangxi Zhuang Autonomous Region (Guangxi Province) in southern China introduced Vi vaccine as a public health tool for school-aged children and for use during outbreaks. Although the reduction in typhoid fever cases from government surveillance has been reported, to date, there has been no formal assessment of the effectiveness of the Vi vaccine use as a public health tool.

Guangxi Province plans to expand the Vi vaccination strategy beyond the school-aged children. An open cluster randomized controlled Vi vaccine effectiveness evaluation was designed and launched in 2002 with the goal of generating policy-relevant data to enable expanded use of Vi vaccine in public health program [8]. Herein, we report initial results on vaccine coverage, safety and logistics of the Vi vaccine when delivered through a mass vaccination campaign that targeted a population aged 5–60 years in a city in Guangxi Province.

## Methods

### Study site and design

**Figure 1:**
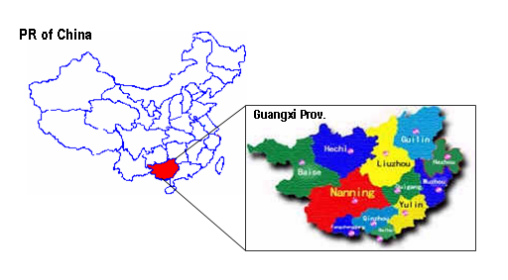
**Hechi in Guangxi Province, PR China**. shows the location of Hechi and Guangxi in China.

The vaccination campaign was carried out in Hechi Prefecture (figure 1), Guangxi Province, between April 8 and May 12, 2003. The study area (referred to in the text as Hechi) is located 420 km northwest of the provincial capital, Nanning, and includes Jin Cheng Jiang (urban) and Don Jiang (rural), the two most populous areas in the prefecture. Agriculture is the major source of income in this subtropical area. The average household annual income is 1,700 RMB (1$ US = 8.3 RMB). Some 80% of the population is of the Zhuang ethnic group. Birth and death rates were 8% and 6%, respectively, in 2001.

In recent years national child immunization program coverage has been over 90% in Hechi, which has a variety of health facilities (85 in total) including hospitals, health centers, factory clinics and private clinics. The Hechi Center for Disease Control (CDC) is responsible for the collection of data on notifiable infectious diseases. In this area, in 1995–1999, typhoid fever incidence rates were 27–153 per 100,000 residents [9] and typhoid fever affected both school-aged children and adults.

The target population for the Vi vaccination campaign was 118,588 persons aged 5–60 years who were registered in the project census conducted in January 2003. The study area was divided into 107 geopolitical clusters, each resembling a unit to be targeted in a public health program. The mean cluster size was 1,103 persons (range: 319–2,610). Of the clusters, 77 with 85,815 persons were in urban areas and the remaining 30, with 32,256 persons, in rural areas. Of households within the clusters, the majority (41%) lived in factory compounds, 21% in private homes, 6% in boarding schools, 6% in government housing and 1% in health institutions.

Clusters were randomly allocated to receive either Vi vaccine or a group A meningococcal (menA) vaccine, the active control. Details of the rationale and design of this open-cluster randomized-effectiveness trial are provided elsewhere [10], [11]. All 107 clusters were stratified according to population size (large ≥ 1,000 or small) and setting (urban or rural) before randomization. The exclusion criteria were pregnancy, breastfeeding, fever >37.5°C at the time of vaccination and presence of severe chronic diseases. The last exclusion criterion is applied to all vaccines in China.

Both vaccines used for this study are licensed and commercially available in China; however, due to the nature of research aspects in determining the effectiveness of the Vi vaccine, ethical and research clearance was obtained from the Guangxi ethical review board, the International Vaccine Institute's institutional review board and the ethical committee of the World Health Organization (WHO). Trial information was disseminated at the community level (meetings and media) and consent was recorded individually on the day of immunization.

### Mass vaccination campaign

Promotional activities were initiated 2 months prior to immunization and included meetings with community leaders and campaign advertisements via local television, loudspeakers, posters and information flyers. The mass vaccination campaign was carried out in 4 stages. Stage I (i.e., the pilot phase) vaccinated a few clusters in order to test and fine-tune the system and to provide final training for the other teams. The next stage was the large-scale vaccination at pre-defined vaccination posts in each cluster according to a pre-set schedule. In stage III, the initial mop-up, the vaccination team re-visited its corresponding vaccination post at the cluster for 1 day. The final mop-up (stage IV) was at a centralized vaccination center (Hechi CDC) after the study population was again invited to be vaccinated.

Both the Vi and the menA vaccines are licensed. They are produced by Lanzhou Institute of Biological Products and administered in 1-dose regimes. A Vi vaccine vial contains two 0.5-ml doses (each with 30 μg of the purified Vi of *Salmonella enterica *subspecies *enterica *serovar Typhi) for both children and adults. This vaccine is administered intramuscularly. The menA vaccine vial contains five freeze-dried 0.2-ml doses (each is 30 μg of the purified group A PS of *Neisseria meningitidis*). Each dose must be reconstituted with 1 ml of PBS (diluent provided with the vaccine) before subcutaneous injection.

Guangxi and Hechi CDC personnel planned, coordinated and launched the mass campaign. Training in good clinical practices (GCPs) was given to all vaccination team members and supervisors 1 month prior to the campaign. The vaccines were delivered by methods that simulated public health program conditions. One site was selected in each cluster (school, health facility, factory or locations such as intersections and squares) to become the vaccination posts. Table 1 depicts the type of personnel involved, responsibilities and numbers needed during the campaign.

**Table 1:** Human resources involved in the Hechi, China, typhoid fever vaccine campaign 2003

**Personnel**	**Function**	**Number**
Vaccine team (30 teams)*		

Leaders (physicians)	Overall responsible for cluster vaccination; daily collection of vaccines and supplies; treat SAE	30
Nurses	Vaccinator	43
Other health workers	Recorder	24
Non-health workers	Recorder	9
Community helpers	Facilitated immunization process; liaison between community and Hechi Center for Disease Control	78
**Other**		

Storage room	Maintained cold chain	4
Data management	Data entry of vaccine records	6
Field health workers	SAE 3-day home visit	3
Drivers		3
Supervisors	Assured adherence to standard operating procedures	7

NOTE: The research-component of the project is not included

* Each team had a leader, vaccinator, recorder and community helper.

Each team was expected to vaccinate about 200 persons each day and to cover 3 or 4 clusters in 20 days. Identification cards similar to those used in the local expanded program of immunization (EPI) were distributed to each household 1 month before vaccination. The cards had identifying information and a study number that corresponded to the household's date and time of vaccination. In order to avoid errors in vaccine allocation, each vaccine team delivered only one vaccine. Those registered in the project census were vaccinated and recorded. However, persons not registered in the project census were offered menA vaccine at the end of the campaign.

Each vaccination team was equipped with: 1 or 2 cold boxes, injecting material, emergency kits, 1 safety disposal box and stationary. All eligible participants were listed in a vaccination record book available at the vaccine posts. Those who came for immunization and gave informed consent were assessed for eligibility by the vaccination team and vaccination status, regardless of received or not received, was recorded in the vaccination record book, which was later entered into a database. Vaccination coverage was calculated from this database.

All aspects of safety for each of the study vaccines were emphasized and the vaccination teams received intensive training. Two particular aspects were monitored: safe injection practices and adverse events (AE). Proper vaccine administration (intramuscular for typhoid Vi and subcutaneous for menA), aseptic injection technique and injection safety (not recapping and disposing of used needles into safety boxes) were closely monitored by supervisors and external observers. Team members were instructed to report all accidents involving sharps and needles to their supervisor. The complete incineration of used safety boxes was monitored by the Hechi CDC staff.

Safety of the vaccines was monitored systematically. All vaccinees were asked to remain in the vaccination posts for at least 15 minutes after vaccination to be monitored for any immediate serious adverse event (SAE) by the physician of the vaccination team. 535 randomly chosen vaccinees were visited for 3 consecutive days after vaccination by the project personnel for solicited adverse event surveillance. Unsolicited (passive) adverse event surveillance was carried out for one month following vaccination by having all healthcare facilities in the study area to report signs and symptoms of patients with history of receiving the vaccine, or hospitalization cases of the vaccinees to Hechi CDC. Physicians attending the patients were asked to fill in a form for all the cases with causality specified. Inadvertent vaccination of pregnant women was to be reported passively during the vaccination campaign; these cases would be followed until delivery. Deaths of study participants are being ascertained through established mortality surveillance by Hechi CDC, which gathers information from death certificates and cremation and hospital records for the 2-year follow-up period. Project personnel reviewed all incoming forms to determine the causality and when unclear, a trial clinical monitor assisted in reviewing the forms.

For both vaccines the manufacturer recommends storage temperatures of 2–8°C. All supplies, including the vaccines, were stored at the logistic hub at Hechi CDC: a 3 × 12 m^2 ^room equipped with 8 refrigerators and 1 freezer. The number of vaccine vials and supplies distributed to each team was recorded daily to calculate vaccine usage and wastage. The maintenance of the cold chain was verified at all stages from the time the vaccine left the manufacturer until it reached the vaccination posts. Maximum/minimum (max/min) thermometers and battery-automated temperature recorders were used to monitor the vaccine temperature during transportation and storage. Regular thermometers in cold boxes were used at the vaccine posts. Temperatures were documented daily in temperature charts – twice for stored vaccines and 3 times for the cold boxes.

## Results

The vaccination campaign lasted 31 workdays, during which 53 clusters received typhoid Vi vaccine and 54 the menA PS vaccine. Thirty teams gave immunizations on an average of 23 days, covering a target population of 118,588 in 107 clusters. Overall, 96,504 people came to the vaccination posts and 92,476 were immunized, yielding an overall vaccine coverage of 78%. In all, 25,605 persons were not vaccinated: 80% (20,472) did not appear for immunization. Table 2 summarizes reasons for not being vaccinated. 80% of the non-immunized did not show-up and 13% were excluded at time of vaccination (serious illness, lactation, pregnancy or fever).

**Table 2:** Reasons for not being vaccinated during the vaccine campaign

Reason	Pregnancy	Lactation	Fever	Serious disease	Refusal*	Did not show-up	Vaccinated previously	Total
No. person	533	714	143	1,851	788	20,472	1,104	25,605
(%)	2	3	1	7	3	80	4	100

* at the time of seeking consent

**Figure 2:**
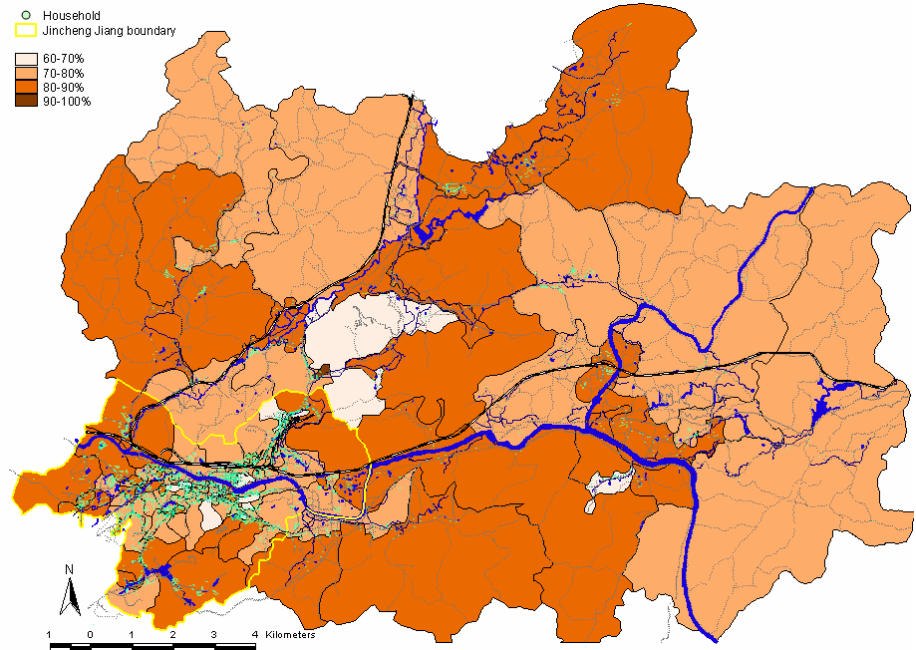
**Vaccine coverage in Hechi**. shows the vaccine coverage by cluster and household locations in Hechi. Darker the color, higher the coverage is. Boundary of the city is also shown.

**Figure 3:**
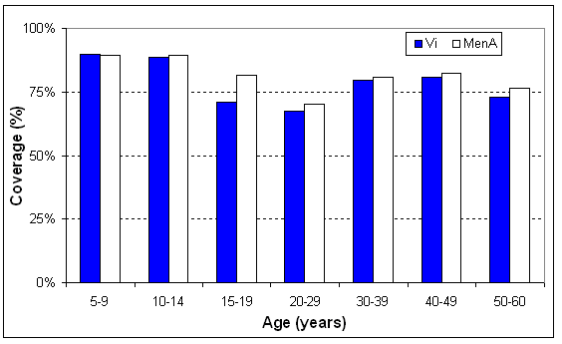
**Vaccine coverage by age group**. shows the coverage of each vaccine by age group.

**Figure 4:**
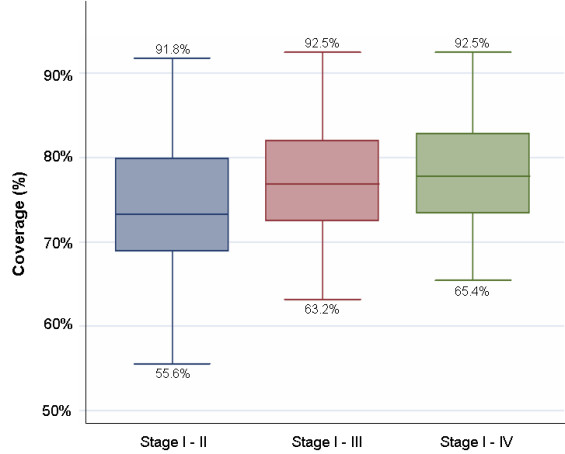
**Cluster vaccine coverage by campaign stages**. shows the cluster vaccine coverage by campaign stages.

The vaccine coverage was 77% and 80% for Vi vaccine and menA vaccine, respectively. Figure 2 shows household locations (mapped by global positioning system technology) and magnitude of coverage by urban and rural area (and by cluster). As shown in figure 3, the highest vaccination coverage (≈90%) was achieved in children less than 15 years of age and was lowest in adolescents (15–19 years) and young adults (20–29 years), reaching 70%. Coverage by gender was similar: 79% in males and 82% in females. High vaccine coverage rates (92.5%) occurred in clusters that corresponded to schools. Figure 4 illustrates vaccine coverage by program stage: 74% (range: 56% – 92%) of the overall coverage was achieved in the first two phases of the vaccination campaign. During mop-up (phases III and IV), 5,269 persons (17% of the total remaining target population) were vaccinated, achieving final coverage of 78% (range: 65% – 93%). Stages I and II lasted 4 days and 5 days (range: 2–9) per cluster, respectively. The initial mop-up stage (stage III) required 4 days and the final mop-up (stage IV) was performed in 3 days. Both of these stages were preceded by 1-day with re-invitation activities.

Three needle-stick injuries were reported during the mass vaccination campaign. All cases happened to the vaccinators of the team. One case occurred during crowded hours at the vaccination post, while two cases occurred due to mishandling of the sharp disposal box. The 3 persons exposed to blood were tested for hepatitis B surface antigen (HBsAg) antibodies and immediately given 1 dose of hepatitis B vaccine according to the local guidelines. No further doses were necessary as all 3 were anti-HBsAg positive.

No life-threatening immediate AE were observed. The surveillance system did identify 1 serious AE; however, this auto-limited febrile case which required hospitalization was not severe and the patient recovered in 12 hours. Of the 413 persons interviewed for solicited AE, 408 were surveyed on all 3 days, and no SAE were reported. In total 129 (38 Vi and 91 menA) AEs determined to be caused by the vaccine were reported from 66 individuals (26 Vi and 40 menA), consisting mainly in fever (21% Vi and 19% menA; ranging between 37.5°C to 39.5°C), malaise (19% Vi and 10% menA), and/or minor rash (19% Vi and 5% menA) around the site of injection. In all, 3,430 hospitalizations were reported 1 month after the immunizations. Of these persons, 300 had been immunized during the campaign. The trial clinical monitor determined that none of the hospitalizations (the majority were scheduled surgeries) were related to vaccine injections.

The Lanzhou Institute initially shipped 60,000 doses of Vi vaccine and 80,000 doses of menA vaccine to the study site by refrigerated (2–4°C) truck monitored by max/min thermometer. Once the temperature reached 14°C for approximately 3 hours. The vaccines were stored in cold rooms at the Guangxi CDC for 6 days where temperature ranged from 2°C to 8°C. On March 29, 2003, the vaccines were sent to the Hechi CDC in a refrigerated truck. This 420-km journey took 6 hours. The cold chain (monitored by battery-powered temperature recorder) was maintained between 4°C and 9°C. At the Hechi CDC, all 140,000 vaccine doses had temperature readings of 2°C to 11°C. The mean temperature recorded in the field was 4.2°C (range: 1.8°C – 12°C). In all, 26 vials of Vi vaccine were discarded due to freezing.

Wastage based on breakage, missing at inventory and unused opened vials was higher as expected for the 5-dose vial of the menA vaccine vial than for the Vi vaccine (12.8% vs. 9.2%., respectively). In conformity with the product information sheet, vials of menA vaccine were discarded within 1 hour of opening if not used. At the end of the campaign the unused doses of both vaccines were shipped to the Hechi CDC headquarters so they could be used elsewhere.

The number of personnel required during the campaign is shown in table 1. The majority were volunteer community members. Transportation from the Hechi CDC to the vaccination posts required a variety of transport: foot, bicycles and motorized vehicles (motorcycles, cars, buses or ambulances).

## Conclusion

The results verify that a mass vaccination campaign with the 1-dose parenteral Vi vaccine or the menA vaccine, which targeted both school-aged children and adults in Hechi, south China, was logistically feasible and safe. An important coverage rate was attained with no major disruption to the normal EPI or other health activities.

In general, there are three main concerns when launching population-based large-scale vaccination campaigns: (1) resources, (2) safety and (3) cost. Especially in developing countries, there is the need to identify resources outside the well-established local vaccine delivery system (usually the EPI program). The Hechi campaign illustrates that an existing healthcare system can deliver vaccines to large populations in a fairly short period of time. Community members can play a key role in the success of promotional activities and delivery of vaccines when an entire city is targeted.

The Hechi campaign monitored and recorded safety systematically and in depth. No life-threatening serious AE were associated with either vaccine. No reported AE (solicited or not) were serious and all patients recovered quickly. Use of safety boxes was introduced for the first time in Hechi during this mass vaccination campaign. Despite instruction on safe handling of sharps, 3 needle-stick injuries were recorded. For future mass vaccination campaigns, including routine EPI activities, safe injection practices must be introduced and emphasized to prevent unnecessary accidents and spread of blood-borne diseases.

A mop-up strategy increased the coverage by 9.6% on average in the lower-coverage clusters and by 0.7% increase in the higher-coverage clusters. Because mop-up strategies imply the need for additional time and costs, this strategy should be planned in advance. Adult coverage was slightly lower than that of children, but a 70% coverage indicates the ability to reach adults during a vaccination campaign. The comprehensive vaccine safety surveillance system, implemented within the existing Hechi health facilities, was useful. Post-licensure monitoring can address many issues regarding safety, such as: uncommon AE, incorrect storage or administration procedures and dissemination of information to the public to maintain confidence in the scheme [12].

The cost-effectiveness of the use of Vi vaccine will be analyzed at the end of the study. However, because the vaccine is locally produced, it seems reasonable to anticipate that unlike EPI vaccines, cost savings can be achieved by reduced transportation costs and in-country production of the vaccine. In an urban area of Vietnam, a locally produced oral cholera vaccine distributed through mass immunization was found to be affordable if introduced in a public health program [13]. In countries, with decentralized health systems, such as China, public health sector introduction or expansion of the use of Vi vaccine can be initiated and financed by local governments.

Mass immunization is considered the most logical control strategy, outside the EPI program, in settings that typically manifest high incidences of disease or in populations exposed to predictable outbreaks [14]. Besides school-based immunization programs, all-age (5–60 years) Vi mass vaccinations are potentially an important public health strategy to prevent typhoid fever in high-risk populations in southern China. Follow-up of the randomized double-blind placebo-controlled trials of Vi vaccine indicate that vaccine protection of 50% lasts for 3 years (manuscript in preparation). The usefulness of Vi vaccine may be further advanced with the development of Vi conjugate vaccine [15]. We anticipate that the vaccine cost results, together with cost-effectiveness analysis of the Hechi program, will complement the above findings and provide objective information to assist policymakers who are increasingly confronted with juggling decisions regarding the use of a growing list of available vaccines while determining health priorities, often under severe budgetary constraints.

## Competing interests

The author(s) declare that they have no competing interests.

## Authors' contributions

YJ, CJA, MA, YH, TP, XZY, AD, DB, JDC have been in part of the conception and design of study, YJ, CJA, SG, ZengJ, LC, LD, RLO, AP, MCD, ZhangJ, ZB, LH, WM, TD, TZ, GJ, JKP, MA, BI, LG, YH, TP, XZY, AD, CMG, DB, JDC compiled, analyzed, and/or interpreted the data, and YJ, CJA, SG, ZengJ, LC, LD, RLO, AP, MCD, ZhangJ, ZB, LH, WM, TD, TZ, GJ, JKP, MA, BI, LG, YH, TP, XZY, AD, CMG, DB, JDC played roles in drafting and/or revising the manuscript.

## Pre-publication history

The pre-publication history for this paper can be accessed here:


